# Durable Self-Cleaning Coatings for Architectural Surfaces by Incorporation of TiO_2_ Nano-Particles into Hydroxyapatite Films

**DOI:** 10.3390/ma11020177

**Published:** 2018-01-23

**Authors:** Enrico Sassoni, Eros D’Amen, Norberto Roveri, George W. Scherer, Elisa Franzoni

**Affiliations:** 1 Department of Civil, Chemical, Environmental and Materials Engineering (DICAM), University of Bologna, Via Terracini 28, 40131 Bologna, Italy; elisa.franzoni@unibo.it; 2 Chemical Center S.r.l., Via S. Donato 5, 40057 Granarolo dell’Emilia (BO), Italy; eros.damen@chemicalcenter.it (E.D.); norberto.roveri@chemicalcenter.it (N.R.); 3 Department of Civil and Environmental Engineering (CEE), Princeton University, 69 Olden Street, Princeton, NJ 08540, USA; scherer@princeton.edu

**Keywords:** soiling, photocatalytic activity, anatase, marble, calcium phosphates, cultural heritage, protection, rain, leaching, consolidation

## Abstract

To prevent soiling of marble exposed outdoors, the use of TiO_2_ nano-particles has been proposed in the literature by two main routes, both raising durability issues: (i) direct application to marble surface, with the risk of particle leaching by rainfall; (ii) particle incorporation into inorganic or organic coatings, with the risk of organic coating degradation catalyzed by TiO_2_ photoactivity. Here, we investigated the combination of nano-TiO_2_ and hydroxyapatite (HAP), previously developed for marble protection against dissolution in rain and mechanical consolidation. HAP-TiO_2_ combination was investigated by two routes: (i) sequential application of HAP followed by nano-TiO_2_ (“H+T”); (ii) simultaneous application by introducing nano-TiO_2_ into the phosphate solution used to form HAP (“HT”). The self-cleaning ability was evaluated before and after prolonged exposure to simulated rain. “H+T” and “HT” coatings exhibited much better resistance to nano-TiO_2_ leaching by rain, compared to TiO_2_ alone. In “H+T” samples, TiO_2_ nano-particles adhere better to HAP (having flower-like morphology and high specific surface area) than to marble. In “HT” samples, thanks to chemical bonds between nano-TiO_2_ and HAP, the particles are firmly incorporated in the HAP coating, which protects them from leaching by rain, without diminishing their photoactivity and without being degraded by them.

## 1. Introduction

**Figure 1:**
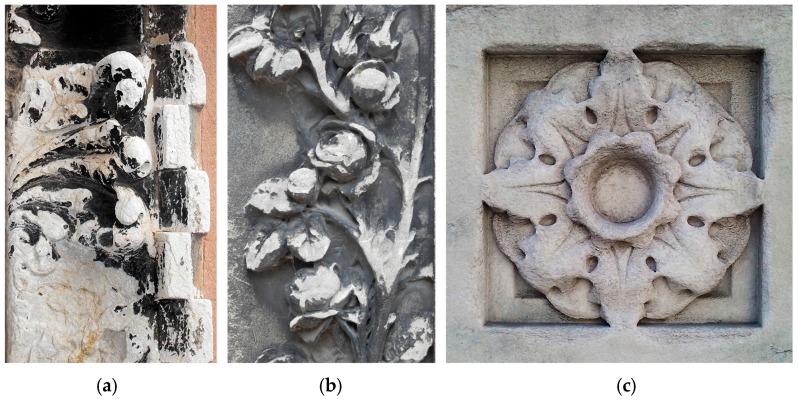
Examples of architectural surfaces affected by soiling: (**a**) Venice, portal of a church (XVIII cent.); (**b**) Paris, tombstone in the *Père Lachaise* cemetery (XIX cent.); (**c**) New York City, decoration in the façade of the New York Public Library (XX cent., cleaned in 2010 and again exhibiting soiling).

Architectural surfaces exposed outdoors are subject to soiling, i.e., darkening originated by accumulation of particulate matter, mainly fine carbonaceous particles rich in dark elemental carbon (Figure 1) [1]. In the case of marble and other carbonatic substrates, darkening can occur by two processes: (i) particles can simply deposit over the surface, by gravity and/or electrostatic attraction; (ii) particles can be embedded in a layer of gypsum formed over marble surface by sulfation caused by atmospheric SO*_x_* [2]. In the latter case, the so-called “black crusts” are formed [3]. While particles simply deposited over the surface can be removed by dusting and/or rinsing with water, cleaning black crusts requires more sophisticated techniques, such as the use of chemicals (e.g., EDTA (eathylene diamine tetraacetic acid)) or laser cleaning [4]. In addition to altering the aesthetic appearance of buildings and monuments, soiling is detrimental for conservation of marble also because it favors sulfation [1] and thermal degradation [5]. Indeed, because of their darker color and their different thermal expansion coefficient, compared to the substrate, dark layers can enhance marble microcracking (the so-called “sugaring”), leading to grain detachment and loss [5,6].

The issue of soiling of architectural surface is so significant that in recent years a huge effort has been dedicated to developing policies for reduction of atmospheric pollutants, not only to reduce human health risks, but also to reduce soiling of building materials. For instance, the European Commission funded several research programs aimed at identifying “tolerable” pollutant concentrations, responsible for “tolerable” soiling of building materials, so that cleaning would be necessary only after an acceptable number of years [7,8]. However, even if the adopted policies for traffic regulation succeeded in reducing SO*_x_* and elemental carbon concentrations, soiling is expected to remain a major problem in the future, because diesel soot and organic carbon will cause a shift from traditional “black soiling” to “yellow-brown soiling” [9,10].

Alongside policies for pollutant regulation, a promising route for reducing soiling of architectural surfaces is to provide building materials with the so-called “self-cleaning ability”. This refers to the ability to prevent soiling and remain clean, thanks to the photocatalytic activity of their surface. Photocatalytic materials are able to oxidize contaminants and bio-organisms, thanks to the redox reactions that take place on their surface when electron/hole pairs, generated by light irradiation, migrate to the surface [11]. Among photocatalytic materials, TiO_2_ (especially in the mineral form of anatase) has been extensively studied and used, because it has the most effective photoactivity, the highest stability and the lowest cost among semiconductor metal oxides [11]. In the case of newly constructed buildings, the photocatalytic activity of TiO_2_ has been exploited by adding nano-particles to paints [12], mortars [13], tiles [13] and glasses [14], to achieve self-cleaning, anti-microbial and air-purifying functionalities.

In the case of historic buildings, the application of TiO_2_ nano-particles over the surface of existing materials was first attempted by applying nano-TiO_2_ colloidal suspensions directly to architectural surfaces [15,16,17,18,19,20,21,22,23,24,25]. After solvent evaporation, TiO_2_ nano-particles are retained on the surface, which acquires self-cleaning ability and remains hydrophilic or even becomes super-hydrophilic [26]. While the efficiency and the compatibility of nano-TiO_2_ suspensions have been extensively investigated, their durability has received attention only recently. In fact, because the bonding to the substrate is only physical in nature, the issue of TiO_2_ nano-particle leaching by rain arises [25,26]. Laboratory studies aimed at assessing the durability of stones treated with TiO_2_ nano-suspensions showed that particle removal by simulated rain is a major problem, leading to a drastic decrease in the photocatalytic activity [20,21,26,27]. The few studies performed on site confirmed that, after prolonged exposure in the field, nano-TiO_2_ is removed by rain, as it is not sufficiently attached to the substrate [22,27]. In addition to the decrease in efficiency, nano-TiO_2_ removal also leads to risks for environmental safety and human health [28]. To prevent leaching of nano-TiO_2_ by rain, the incorporation of TiO_2_ nano-particles into coatings of different nature has been proposed. The investigated routes include inorganic coatings (e.g., silica [29]), organic coatings (e.g., acrylic [30,31], fluorinated [31,32] and silane polymers [33]) and hybrid coatings (e.g., silica and polydimethylsiloxane [34,35]). In this way, multi-functional coatings can be obtained, which combine the self-cleaning ability with water repellency and mechanical strengthening [29,30,31,32,33,34,35]. However, in the case of organic coatings, the durability of the polymer-TiO_2_ composites has been found to be at risk, because TiO_2_ nano-particles themselves catalyze polymer degradation [30,32].

In this study, we investigated the combination of TiO_2_ nano-particles with hydroxyapatite as an innovative route to provide marble with durable self-cleaning functionality, simultaneously with mechanical strengthening. Hydroxyapatite coatings were proposed a few years ago for protection of marble from dissolution in rain [36,37,38,39,40] and for mechanical consolidation of weathered marble [41,42,43] and limestone [44,45,46,47,48,49,50,51]. The idea is to form hydroxyapatite (HAP, Ca_10_(PO_4_)_6_(OH)_2_) by reacting the stone with an aqueous solution of diammonium hydrogen phosphate (DAP) [44]. By reaction between the PO_4_^3−^ ions coming from DAP dissociation in water and Ca^2+^ ions coming from millimolar dissolution of the substrate [44] and/or externally supplied [36], HAP is expected to form. Besides HAP, which is the most stable calcium phosphate at pH > 4 [52] and hence the most desirable phase to form, other calcium phosphate (CaP) phases may result from the reaction [44,53]. These CaP phases are metastable and are expected to finally lead to formation of HAP [44]. In any case, as long as CaP phases with solubility lower than calcite are formed, their formation is expected to be beneficial for stone protection and consolidation [36]. Previous studies showed that the features of the CaP coating (in terms of composition, presence of cracks and pores) can be optimized by controlling the DAP concentration [39,44], by adding calcium sources [36] and by adding alcohols to the DAP solution [39,54]. In addition to the good performance assessed in the lab in terms of both protection of marble and consolidation of marble and limestone [54], encouraging results have been obtained also from the first applications onto real monuments [49,55].

Here, we investigated the combination of TiO_2_ nano-particles with the HAP-based treatment with three objectives: (i) preventing nano-TiO_2_ leaching by rain, by incorporation into the HAP coating (which is not expected to be degraded by TiO_2_ photoactivity, unlike polymeric coatings); (ii) enhancing the photocatalytic activity of TiO_2_, thanks to combination with HAP (as suggested by previous studies on HAP-TiO_2_ composites for biomedical applications [56,57,58]); (iii) enhancing the development of HAP layers over the surface and inside cracks, thanks to the presence of TiO_2_ nano-particles (acting as seeds for HAP nucleation [59] and limiting shrinkage during drying [60]).

Two different routes for HAP-TiO_2_ combination were investigated: (i) sequential application of HAP, followed by nano-TiO_2_ (labeled “H+T”); (ii) simultaneous application of the two, by introducing TiO_2_ nano-particles directly into the DAP solution used to form HAP (labeled “HT”). The combined treatments were applied to Carrara marble and compared to application of HAP alone (labeled “H”) and nano-TiO_2_ alone (labeled “T”), as well as to the untreated reference (labeled “UT”). The morphology of the coatings was observed by ESEM/EDS, while their microstructure was evaluated by cutting cross sections by FIB-SEM. The coating composition and, in particular, the possible formation of Ti–O–P bonds were investigated by Raman spectroscopy. The effects of the treatments were characterized in terms of consolidating ability (i.e., increase in marble cohesion measured by ultrasound), aesthetic compatibility (i.e., color change after treatment) and self-cleaning ability (i.e., ability to discolor a methylene blue stain after UV exposure). Finally, the resistance of TiO_2_ nano-particles to leaching by rain was evaluated by subjecting samples to simulated rain by a custom-designed apparatus, which drips water over the samples at a controlled rate and collects the runoff water for analysis. After dripping an amount of water corresponding to the average rain of ~6 years in Bologna (Italy), the residual self-cleaning ability was assessed by carrying out the photoactivity test and by analyzing samples by ESEM/EDS. The amount of Ti in the runoff water, indicative of nano-TiO_2_ removal by rain, was also analyzed by ICP-OES (plasma optical emission spectrometry).

The combined treatments “H+T” and “HT” exhibited much better resistance to nano-TiO_2_ leaching by rain, compared to the “T” treatment. This resulted in higher self-cleaning ability after prolonged exposure to rain and lower Ti amounts detected in the runoff water. In the case of the “H+T” treatment, the positive effect of HAP-TiO_2_ combination can be ascribed to the better adhesion of nano-TiO_2_ to HAP (having flower-like morphology and high specific surface) than to marble. In the case of the “HT” treatment, Raman results suggest formation of chemical bonds between nano-TiO_2_ and HAP. In this way, the nano-particles are strongly bonded to the transparent and durable HAP layer, which protects them from the leaching action of rain, without diminishing their photoactivity and without degradation of the HAP matrix.

## 2. Results and Discussion

### 2.1. Coating Morphology and Microstructure

**Figure 2:**
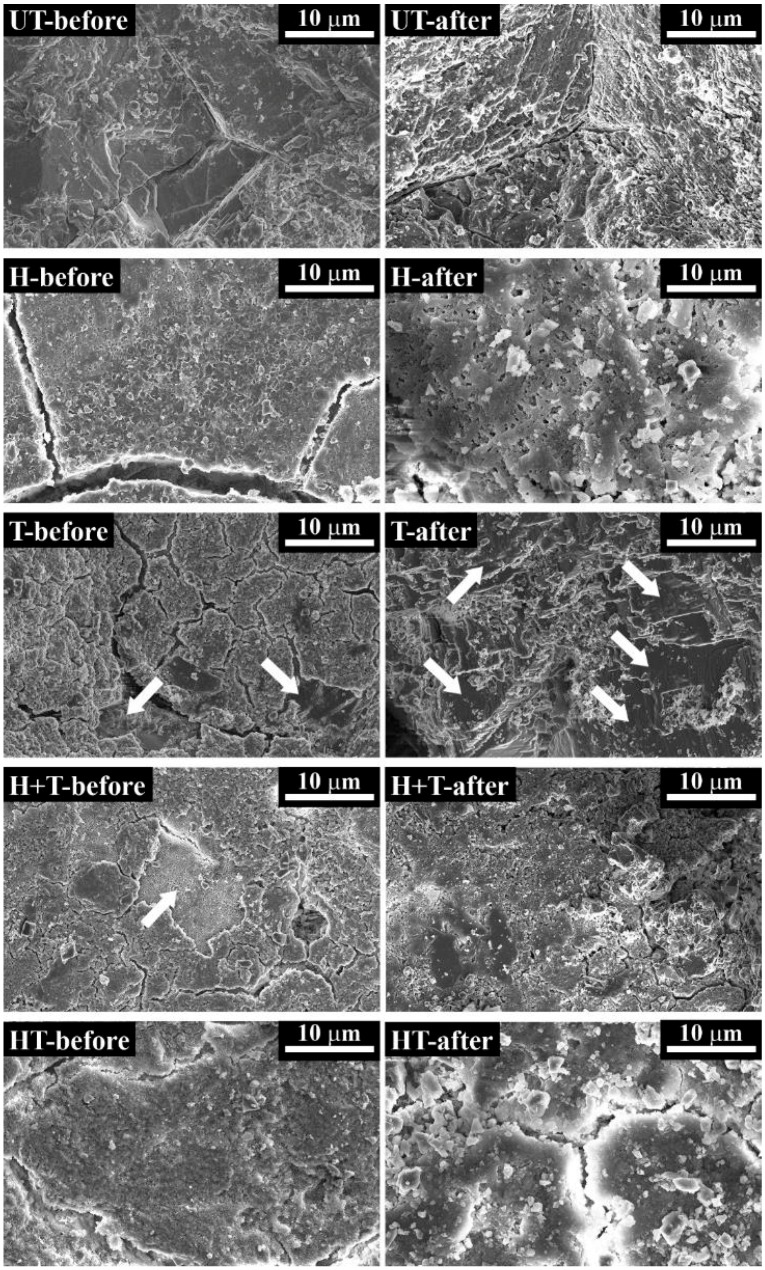
Surface morphology of untreated and treated samples, before and after simulated rain.

**Figure 3:**
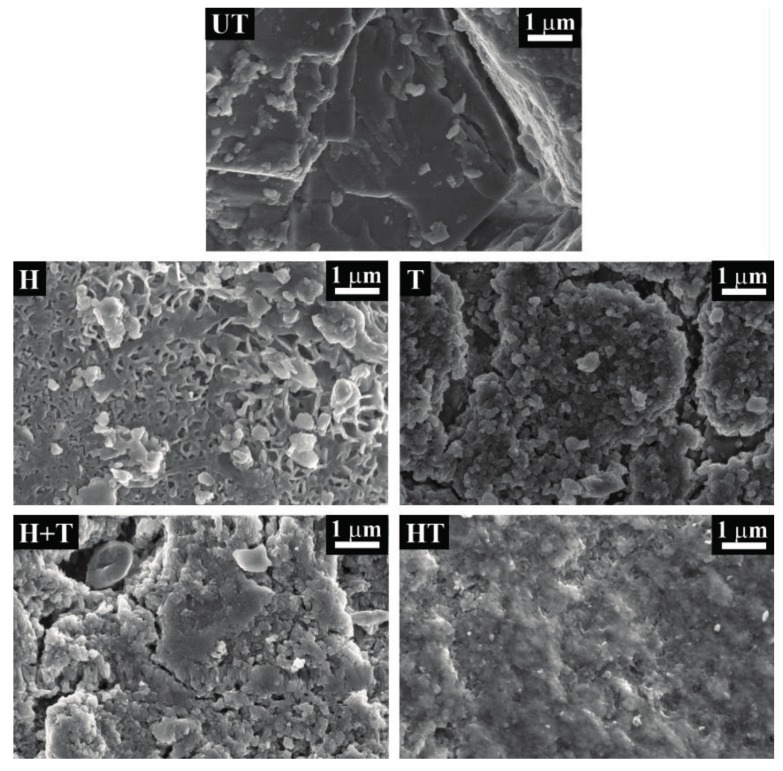
Surface morphology of untreated and treated samples before exposure to simulated rain.

The morphology of untreated and treated samples, before artificial rain, is illustrated in Figure 2 (“-before” condition) and, at higher magnification, in Figure 3.

The “UT” reference exhibits inter-granular micro-cracks, which were produced by subjecting marble to accelerated ageing by heating (cf. Section 3.1), to obtain samples with characteristics similar to naturally weathered marble and suitable for testing the treatments [41,61,62]. After the “H” treatment, a coating with flower-like morphology is formed, which exhibits diffused cracking. Cracks are presumably developed during drying, because the coating thickness exceeds the critical one, below which cracking is thermodynamically inhibited [63]. In the case of the “T” sample, formation of a cracked “crust” of TiO_2_ particles over the surface can be observed. Correspondingly, a very high Ti content is detected by EDS (Energy Dispersive Spectrum) on the observed area (Ti = 31.9 wt. %, Table 1). Notably, cracking causes detachment of the TiO_2_ layer in several areas, leading to exposure of bare marble (indicated by white arrows in Figure 2). Similarly, the “H+T” sample exhibits a cracked surface layer of Ti particles, again leading to high Ti content (Ti = 32.4 wt. %, Table 1). In this sample, in a few areas where the TiO_2_ layer detached from the substrate, the underlying HAP coating can be seen (indicated by the white arrow in Figure 2). In the case of the “HT” coating, less diffused cracking is present (Figure 2) and the coating appears as smoother and denser (Figure 3). In this sample, a much lower Ti content (0.1 wt. %) was detected, compared to the “T” and “H+T” samples (31.9 and 32.4 wt. %, respectively) (Table 1). This is a consequence of the application of a limewater poultice as the last step of the “HT” treatment procedure (cf. Section 3.2). In addition to favoring HAP formation and removing unreacted DAP [64], the limewater poultice also removes all the excess TiO_2_ particles not embedded in the HAP coating.

**Figure 4:**
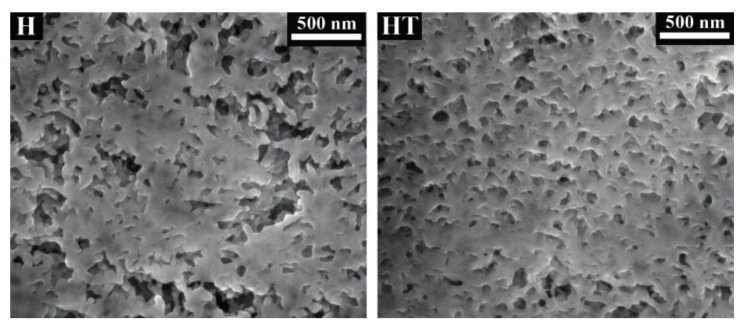
Microstructure of cross sections of “H” and “HT” samples obtained by FIB-SEM.

Besides the smoother and denser surface morphology (Figure 3), the different microstructure of the “HT” coating was confirmed by observation of cross sections obtained by FIB-SEM (Figure 4). Compared to the “H” sample, where relatively coarse pores are present, the “HT” sample exhibits a refined microstructure. This is thought to be a positive consequence of three factors. First, the presence of TiO_2_ nano-particles in the DAP solution is expected to have a positive effect on HAP formation, as nano-particles can provide sites for HAP nucleation by “seeding” effect [59]. Second, the presence of nano-particles can reduce shrinkage during drying, thus reducing formation of cracks and pores, similarly to the case of particle modified consolidants [60]. Third, the presence of isopropanol in the nano-TiO_2_ suspension and, hence, in the doped DAP solution (although in low concentration) is expected to have favorably affected HAP formation. Indeed, alcohol molecules weaken the hydration sphere of PO_4_^3−^ ions in solution, thus making them more reactive for CaP formation [54]. Notably, the Ti content detected by EDS in the cross section of the “HT” sample (0.2 wt. %) is similar to that measured on the surface (0.1 wt. %), suggesting an even distribution of TiO_2_ nano-particles across the whole thickness of the coating (about 4–5 μm). Accordingly, the dispersion of TiO_2_ nano-particles has been reported to be promoted by the presence of HAP [57].

### 2.2. Coating Composition

**Figure 5:**
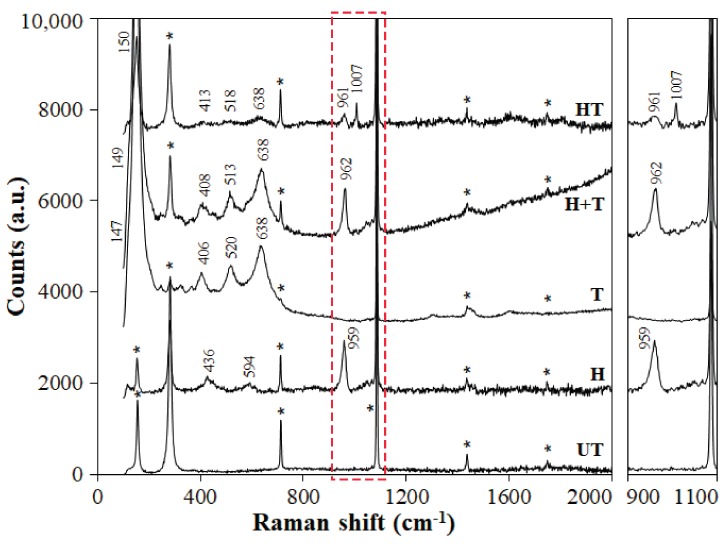
Raman spectra of the various samples and magnification of the 900–1100 cm^−1^ region.

The Raman spectra obtained for the various conditions are reported in Figure 5. In the “UT” sample, all the observed bands (indicated by a star in Figure 5) are owing to calcite [42]. In the case of the “H” treatment, the new bands at 959, 594 and 434 cm^−1^ suggest formation of HAP, although formation of octacalcium phosphate (OCP) cannot be completely excluded [65]. In case (also) OCP was formed, this is not expected to negatively affect the treatment performance, because OCP is slightly more soluble than HAP, but still less soluble than calcite [36]. In the “T” sample, the new bands at 638, 520, 406 and 147 cm^−1^ confirm the presence of anatase on the sample surface [66]. In the “H+T” sample, a clear band owing to HAP (and/or OCP) at 962 cm^−1^ [65] and bands owing to anatase at 638, 513, 408 and 149 cm^−1^ [66] are present. The deviation of the baseline is due to florescence, which is likely owing to the nanometric size of the TiO_2_ nanoparticles. In the “HT” sample, the HAP band at 961 cm^−1^ and the anatase band at 638 cm^−1^ are clearly visible, while shoulders at 518 and 413 cm^−1^ further suggest the presence of anatase. The band at 150 cm^−1^ results from the overlapping of the anatase band at 147 cm^−1^ [66] and the calcite band at 154 cm^−1^ (“UT” sample). The strong reduction in the anatase bands in the “HT” sample is a consequence of the very low amount of TiO_2_ nano-particles used for this treatment (cf. Section 3.2), as also pointed out by EDS results (Table 1). Notably, in the “HT” sample a new band appears at 1007 cm^−1^, as highlighted in the magnified spectrum on the right of Figure 5. This new band is thought to indicate formation of Ti–O–P bonds, which has been reported in the literature in the region around 990 cm^−1^ [67]. However, to conclusively ascertain formation of such a bond, further analyses seem opportune.

### 2.3. Consolidating Ability

The results of the ultrasonic tests to evaluate mechanical strengthening are reported in Table 2. After artificial weathering by heating, the “UT” sample exhibits *UPV* = 0.7 km/s, equal to 23% of the initial value (2.9 km/s before heating). Based on the classification of marble conservation state reported in [68], artificial ageing causes the passage from a condition of “progressive granular disintegration” to a condition of “complete structural damage”. After the “H” treatment, marble cohesion is completely restored and even slightly improved (106% of the initial *UPV* value), thanks to HAP formation inside the micro-cracks, which results in more effective bonding of calcite grains [42]. The “T” sample undergoes basically no mechanical improvement, as expected, because the TiO_2_ nano-particles hardly penetrate into micro-cracks and anyway have no bonding ability. Consistently, the “H+T” sample exhibits a similar improvement as the “H” sample (103% of the initial *UPV*), with no additional benefit deriving from the subsequent application of the “T” treatment. Similarly, the “HT” treatment provides the same mechanical improvement as the “H” treatment, because the DAP solution is able to penetrate deeply into marble micro-cracks, while the TiO_2_ nano-particles tend to accumulate on the surface.

### 2.4. Compatibility

As reported in Table 3, none of the treatments was responsible for an unacceptable color change (ΔE* > 5 [45]), in all cases the color change being below the threshold identified as “just noticeable difference” by human eye (ΔE* = 2.3 [69]). This means that in terms of aesthetic compatibility all the treatments would be suitable for application on white marble (for darker stones, specific tests should be carried out before application). The “H” and the “T” treatments cause similar total color changes (nevertheless, below the level distinguishable by human eye), but they alter the lightness (L*) and the yellow-blue (b*) coordinates in opposite ways. Notably, the combination of the two treatments (“H+T” and “HT”) leads to a lower color change than the two treatments alone, which can be ascribed to the opposite alterations in L* and b* mentioned above.

### 2.5. Self-Cleaning Ability

**Figure 6:**
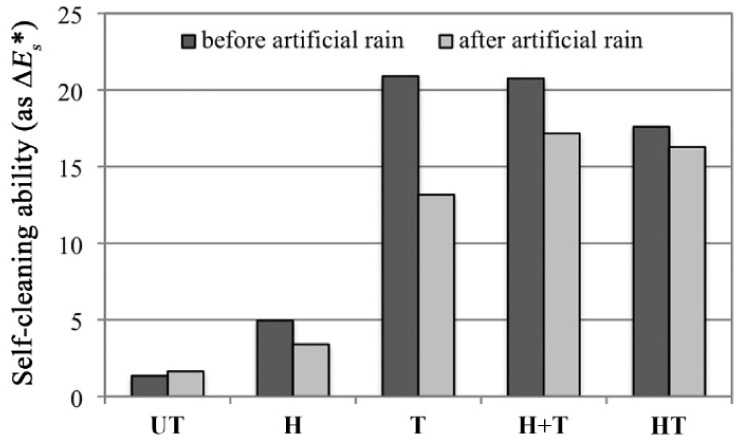
Self-cleaning ability (defined as the ability to cause discoloration of a methylene blue stain, ΔE_s_*) of untreated and treated samples, before and after simulated rain.

The self-cleaning ability of the samples, expressed in terms of discoloration of the methylene blue stain after UV exposure (ΔE_s_*), is shown in Figure 6 (dark grey bars). The untreated reference “UT” undergoes some minimal discoloration (ΔE_s_* = 1.3), consistently with previous studies reported in the literature [29,30], which ascribed discoloration in untreated stone to some weak absorption of UV light by the dye [29]. The “H” sample exhibits some modest discoloration (ΔE_s_* = 4.9, sensibly higher than the untreated reference), which is due to the low photocatalytic activity of hydroxyapatite itself [56,57]. The “T” and “H+T” treatments, which lead to high and comparable amounts of nano-TiO_2_ over the surface (Table 1), exhibit high and comparable discoloration of methylene blue (ΔE_s_* = 20.9 and 20.7, respectively). In the case of the combined “HT” treatment, a slightly lower self-cleaning ability is found (ΔE_s_* = 17.6). Considering that no accumulation of TiO_2_ nano-particles over the surface was found by ESEM (environmental scanning electron microscope) (Figure 2 and Figure 3) and that a very low amount of Ti was detected by EDS (Ti = 0.1 wt. %, Table 1), such self-cleaning ability is very remarkable. The presence of such photoactivity is possible thanks to the transparency of HAP [57], which allows also TiO_2_ nano-particles below the surface to be photoactivated and contribute to the measured photoactivity [57]. Even if a direct comparison between values of stain discoloration obtained in this study and those reported in the literature for alternative treatments is made difficult by the influence of the specific experimental conditions adopted in each study, the self-cleaning ability of the “T”, “H+T” and “HT” coatings investigated here is similar to that reported in the literature (ΔE_s_* = 13–15 [15,29]), which confirms the high potential of the investigated treatments.

### 2.6. Resistance to Simulated Rain

The residual self-cleaning ability after exposure to artificial rain is illustrated in Figure 6 (light grey bars). The “UT” sample undergoes basically no modification (ΔE_s_* = 1.6), consistently with the fact that discoloration in untreated stone only depends on UV absorption by the dye [29]. The “H” sample exhibits a small reduction in the original self-cleaning ability. This might be due to some minor deterioration of the HAP coating after prolonged exposure to simulated rain, as suggested by new small pores visible by ESEM on the coating (Figure 2, “-after” condition). In the case of the “T” sample, a significant loss of self-cleaning ability occurred (from ΔE_s_* = 20.9 to ΔE_s_* = 13.1, with a reduction of −37%). This is due to diffused removal of the TiO_2_ “crust” formed over the surface after treatment, as clearly indicated by ESEM images of samples subjected to simulated rain (Figure 2). Numerous bare areas, indicated by the white arrows in Figure 2, are present and the Ti content detected by EDS over the sample surface is drastically reduced after the simulated rain (from 31.9 to 1.1 wt. %, Table 1). The removal of TiO_2_ nano-particles from the surface is also confirmed by detection of Ti in the runoff water collected after dripping over the “T” sample and analyzed by ICP-OES (Table 1). Compared to the “T” sample, the “H+T” sample exhibits a lower decrease in the self-cleaning ability (from ΔE_s_* = 20.7 to ΔE_s_* = 17.1, with a reduction of −17%). Consistently, almost no removal of TiO_2_ nano-particles is visible in the ESEM images (Figure 2), no reduction in Ti is detected by EDS (Table 1) and a minor concentration of Ti in the runoff solution is found by ICP-OES (Table 1). The good resistance of the “H+T” coating to leaching by rain seems to be ascribable to the better physical adhesion between the TiO_2_ particles and HAP, compared to marble. This better adhesion is probably favored by the flower-like morphology of the HAP coating and the high specific surface area of the HAP crystals (Figure 3). The “HT” samples exhibit the lowest decrease in self-cleaning ability (from ΔE_s_* = 17.6 to ΔE_s_* = 16.3, with a reduction of only −7%) (Figure 6). This is possible thanks to nano-TiO_2_ incorporation into the HAP coating, with formation of chemical bonds between the particles and the coating, as suggested by Raman results. Thanks to such chemical bonding and to the durability of the HAP coating to simulated rain (Figure 2), the Ti content after exposure to rain is similar to that before (Table 1) and only a minimal Ti content is detected in the runoff solution (Table 1).

The high potential of the combined “H+T” and “HT” treatments is hence confirmed, as they both are able to resist nano-TiO_2_ leaching by rain and to preserve their photocatalytic activity much better than the “T” treatment. Because simple application of nano-TiO_2_ dispersions to marble surfaces (like the “T” treatment) is currently already carried out in the field, the innovative treatments investigated in this study exhibit clear advantages.

## 3. Materials and Methods

### 3.1. Marble

Prismatic samples (30 × 30 × 20 mm^3^) were sawn from a freshly quarried slab of Carrara marble (supplied by Imbellone Michelangelo s.r.l., Bologna, Italy). Prior to treatment application, samples were subjected to artificial weathering by heating at 400 °C for 1 h, according to a previously developed method [61,62]. This weathering procedure effectively reproduces in laboratory conditions the degradation state of marble exposed outdoors, consisting in intergranular micro-cracking [41].

### 3.2. Treatments

After artificial weathering, part of the samples was left untreated (“UT”), while the other samples were subjected to the following treatments:

- “H”, consisting in the HAP treatment alone. A 3M aqueous solution of DAP (Sigma-Aldrich, Milan, Italy, assay ≥ 98%, reagent grade) was applied by brushing until apparent refusal (8 brush strokes). Then, samples were wrapped in a plastic film to avoid evaporation and left to react for 48 h. After removal of the plastic film and rinsing with deionized water, samples were left to dry at room temperature for 4 days. Afterwards, a poultice of so-called limewater (i.e., a saturated solution of calcium hydroxide) was prepared using cellulose pulp (MH300 Phase, Italy) and Ca(OH)_2_ (Sigma-Aldrich, reagent grade), with a limewater:cellulose pulp weight ratio of 6:1. The limewater poultice was applied with the twofold aim of (i) supplying additional calcium ions for reaction with unreacted DAP and (ii) removing unreacted residues at the end of the treatment, by drying in contact with the samples [64]. A sheet of Japanese paper was inserted between the samples and the poultice to avoid sticking. After covering with the poultice, samples were wrapped in a plastic film for 24 h, then the film was removed and the poultice was left to dry in contact with the samples until constant weight. After removal of the dried poultice, samples were rinsed with deionized water and finally left to dry at room temperature.
- “T”, consisting in the TiO_2_ treatment alone. A 2 wt. % suspension of TiO_2_ particles (98.1% anatase, 1.9% brookite, crystallite average size between 10 and 20 nm) in 80-20 wt. % water-isopropanol medium was applied by a single brush stroke. A maximum concentration of 2 wt. % was recommended in the literature to avoid particle agglomeration [24].
- “H+T”, consisting in the application of treatments “H” and “T” in sequence, applied exactly as described above for the single treatments.
- “HT”, consisting in the application of a single treatment combining HAP and nano-TiO_2_. The combined treatment was obtained by mixing the 2 wt. % TiO_2_ suspension and the 3M DAP solution in the weight proportion 1.5:98.5, respectively. This proportion was selected based on the number of brush strokes adopted for treatment application: 8 strokes in the case of “HT” (like the “H” samples), instead of a single stroke in the “T” samples. Because repeated application leads to particle accumulation over the surface, a lower particle concentration than in the “T” treatment was selected. After treatment application by brushing and after reaction for 48 h wrapped in a plastic film, samples were dried at room temperature and then subjected to application of the limewater poultice, as described for the “H” samples. After drying of the poultice, samples were finally rinsed with deionized water and dried at room temperature.

### 3.3. Characterization

#### 3.3.1. Coating Morphology and Microstructure

Sample morphology was assessed by observation with an environmental scanning electron microscope (FEI Quanta 200 FEG ESEM, Hillsboro, OR, USA), equipped with an energy dispersive X-ray spectroscopy device (Oxford Instruments EDS probe, Concord, MA, USA). Samples were coated with carbon before observation to make them conductive (observations were carried out in high vacuum for better resolution).

The microstructure of the “H” and “HT” coatings was analyzed by cutting a cross section of the samples (made conductive by carbon coating), using a focused ion beam SEM (FEI StrataTM DB 235 FIB, Hillsboro, OR, USA). After cutting the cross sections, samples were coated for a second time, to make the cross section conductive, and then observed using the FEI Quanta 200 FEG ESEM.

#### 3.3.2. Coating Composition

The composition of the coatings and, in particular, the possible formation of Ti-O-P bonds in the combined treatments was investigated by Raman spectroscopy, using a Renishaw Raman Invia spectrometer (Renishaw, Turin, Italy), linked to a Leica DMLM optical microscope (Leica, Germany). For each treatment, at least three spectra were acquired in different positions.

#### 3.3.3. Consolidating Ability

The consolidating ability was evaluated by measuring the ultrasonic pulse velocity (*UPV*) across the samples, before and after treatment. *UPV* is a suitable parameter for assessing the conservation state of marble, because it is very sensitive to the opening of micro-cracks caused by weathering and their sealing caused by consolidants [68,70]. Measurements were carried out across the 20 mm side of the samples (9 replicates for each condition), using a Matest instrument with 55 kHz transducers and inserting a rubber couplant between the samples and the transducers.

#### 3.3.4. Aesthetic Compatibility

To ensure that marble aesthetic appearance does not undergo any unacceptable alteration after treatment [45], the color change caused by the treatments was assessed by comparing untreated and treated samples. The color parameters in the CIELAB color space (L* = black ÷ white; a* = red ÷ green; b* = yellow ÷ blue) were determined using an optical fiber spectrophotometer Ocean optics USB2000+ in reflective mode. The color change ΔE* was then calculated according to the formula ΔE* = [(Δa*)^2^ + (Δb*)^2^ + (ΔL*)^2^]^1/2^, as the average for 5 measurements.

#### 3.3.5. Self-Cleaning Ability

According to a method widely adopted in the literature (e.g., [19,29,30]), the self-cleaning ability was evaluated as the capability to catalyze discoloration of a stain on the marble surface, after UV exposure for a certain time. Untreated and treated samples were first stained by application of a drop (110 mL) of a methylene blue solution (10 g/L). After drying, the color parameters of the stained samples were determined as described in Section 3.3.4. Stained samples were exposed to UV radiation (50 W, λ = 365 nm, distance lamp/samples 20 cm) for 2 h. This exposure time was regarded as sufficient, considering that most of the total discoloration is experienced during the first few hours of exposure [29]. The color parameters were remeasured and the stain discoloration ΔE_s_* was calculated as the color change between the conditions before and after UV exposure, using the formula reported in Section 3.3.4. ΔE_s_* was calculated as the average for 5 points.

#### 3.3.6. Resistance to Simulated Rain

**Figure 7:**
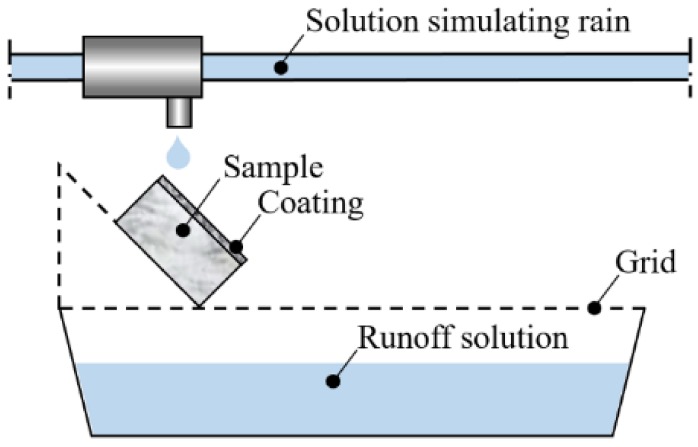
Custom-designed set-up for simulating rain.

The permanence of the self-cleaning ability after leaching by rain was evaluated by subjecting untreated and treated samples (not previously used for any other test) to simulated rain and then performing the stain discoloration test as described in Section 3.3.5. The custom-designed apparatus sketched in Figure 7 was used to drip deionized water over the samples, tilted by 45° with respect to the horizontal (two drops per sample). Deionized water at pH 5.6 (representative of clean rain) was used, considering that the dissolution kinetics of calcite is not expected to vary significantly within the pH range between ~5 and ~5.6 [37,71]. Water at pH < 4 was not used, because such an acidic value would not be representative of current rain pH in Europe, which is ~5 on average [72]. Ten cycles of dripping for 2 h at a rate of 420 mL/h, followed by drying at room temperature for 22 h, were carried out. The total volume of water dripped onto each sample corresponded to the volume of rain that falls onto the same area in ~6 years in Bologna, Italy (average rainfall 80 cm/year). Two samples for each treatment were subjected to the simulated rain. After contact with the samples, the runoff water was collected and analyzed by inductively coupled plasma optical emission spectrometry (ICP-OES), using a Horiba ULTIMA 2 spectrometer (Horiba, France), to determine the Ti concentration (indicative of nano-TiO_2_ removed from sample surface). Possible alterations in the coating morphology were evaluated by ESEM/EDS, performed as described in Section 3.3.1.

## 4. Conclusions

In this study, the combination of TiO_2_ nano-particles and hydroxyapatite (HAP) was investigated as a possible route to provide marble with durable self-cleaning ability and mechanical strengthening at the same time.

When TiO_2_ nano-particles are applied after formation of a HAP coating over the marble surface (“H+T” samples), a better resistance to nano-TiO_2_ leaching by simulated rain and, hence, more durable self-cleaning ability are obtained, compared to nano-TiO_2_ application directly onto marble surface. This is thought to be a consequence of the better adhesion of TiO_2_ nano-particles to HAP, having flower-like morphology and high specific surface area, compared to marble.

When TiO_2_ nano-particles are introduced directly into the aqueous phosphate solution used to form HAP (“HT” samples), the resulting coating exhibits a refined microstructure, presumably thanks to the seeding effect of TiO_2_ nano-particles and their restraining effect against shrinkage during drying. The self-cleaning ability of the combined “HT” treatment exhibits much higher durability than the other conditions, with a minimal decrease of the self-cleaning ability after prolonged exposure to simulated rain and a minimal release of TiO_2_ nano-particles, with a highly positive effect also in terms of environmental and human safety. This is possible thanks to formation of chemical bonds between the particles and the HAP coating. In this way, TiO_2_ nano-particles are firmly incorporated in a transparent and durable HAP coating, which protects them from leaching by rain, without diminishing their photoactivity and without being degraded by them.

Considering the much better durability of the combined coatings investigated in this study, compared to simple application of nano-TiO_2_ dispersions directly to marble surfaces (which is currently the most common practice in the field), the advantage of the new treatments is evident. Further tests to assess the durability of the new coatings in the field will be the next step of the research.

## Figures and Tables

**Table 1:** Ti concentrations determined in samples analyzed by ESEM/EDS, before and after the simulated rain, and in the respective runoff water analyzed by ICP-OES. The EDS contents were determined on the whole areas illustrated in Figure 2.

Sample	Ti wt. % (EDS)		Ti ppm (ICP)
	Before Rain	After Rain	Runoff Solution
T	31.9	1.1	0.256
H+T	32.4	33.1	0.085
HT	0.1	0.1	0.025

**Table 2:** Variations in ultrasonic pulse velocity (*UPV*) after treatment (values are averages for 9 replicates, standard variations in brackets).

Sample	*UPV* (km/s)	% of Initial *UPV* *
UT	0.7 (±0.1)	23
H	3.1 (±0.2)	106
T	0.7 (±0.1)	24
H+T	3.0 (±0.5)	103
HT	3.1 (±0.2)	106

* Unweathered marble (*UPV* = 2.9 km/s) was taken as reference (100% of initial *UPV*).

**Table 3:** Variations in color parameters after treatment (ΔL* = change in the black ÷ white coordinate; Δa* = change in the red ÷ green coordinate; Δb* = change in the yellow ÷ blue coordinate; ΔE* = total color change. Values are averages for 5 replicates).

Sample	ΔL*	Δa*	Δb*	ΔE*
H	1.83	−0.35	−2.25	2.18
T	−0.97	−0.24	0.60	1.94
H+T	1.21	−0.23	−1.50	1.25
HT	−0.19	−0.35	−1.72	0.64
